# “It is like living in a diminishing world”: older persons’ experiences of living with long-term health problems – prior to the STRENGTH intervention

**DOI:** 10.1080/17482631.2020.1747251

**Published:** 2020-04-10

**Authors:** Cecilia Åberg, Catharina Gillsjö, Jenny Hallgren, Mia Berglund

**Affiliations:** a School of Health Sciences, University of Skövde, Skövde, Sweden; b School of Health and Welfare, Jönköping University, Jönköping, Sweden; c College of Nursing, University of Rhode Island, Kingston, RI, USA

**Keywords:** Ageing, health problem, illness, multimorbidity, patient perspective, phenomenology, qualitative research, reflective lifeworld research, strength, well-being

## Abstract

**Introduction**: Ageing is often associated with multiple long-term health problems influencing older persons’ well-being in daily living. It is not unusual that the point of interest in research is often on the management of the actual health problem instead of being holistic and person-centred.

**Purpose**: To describe the phenomenon of living with long-term health problems that influence daily living, from the older persons’ perspective.

**Methods**: Qualitative individual interviews were conducted with 34 older persons living with long-term health problems. The data were analysed using a Reflected Lifeworld Research (RLR) approach, grounded in phenomenology. Results: Life with long-term health problems entails living in a diminishing world. It entails living in uncertainty, not being able to trust one’s own ability. The freedom to make decisions of your own is deprived by relatives and health-care providers. Living with long-term health problems entails being dependent on support in daily life and a strive to maintain meaningfulness in daily living.

**Conclusions**: The results address a need for extended individual and holistic guidance and support in living with long-term health problems to increase the older person’s sense of well-being and meaning in life.

## Introduction

The rapid acceleration of a worldwide ageing population is a challenge for healthcare systems since ageing is often accompanied by a variety of health problems. These health problems are often both multiple and complex and associated with difficulties and disabilities in daily living (Cavanaugh & Blanchard-Fields, 2011; Fratiglioni et al., 2010; GBD 2015, DALYs and HALE Collaborators, 2016; Marengoni et al., 2016; Summer Meranius & Josefsson, 2017; WHO, 2006, 2013, 2015). Despite the large numbers of older persons with long-term health problems, the provision of healthcare not as comprehensive as is needed (WHO, 2015), furthermore, globally, health improvements in the ageing population are not generally the focus of interest (Chang et al., 2019). Older persons living with health problems in Sweden are recognized by Swedish Agency for Health Technology Assessment and Assessment of Social Services (SBU, 2017a) as a population in which there is a gap of scientific knowledge and a need to carry out and strengthen practice-oriented research. The majority of available studies involving older persons and their health problems have a focus on the management of the health problems per se, or on the coexistence of a relatively small number of health problems, rather than a holistic orientation on the health problems affecting older persons’ daily living in their context. This predominant focus can be problematic since many of the older persons’ health problems are interrelated and occur simultaneously (Chang et al., 2019; Marengoni et al., 2011; SBU, 2017a; Summer Meranius, 2010; Summer Meranius & Josefsson, 2017; WHO, 2015). In order to promote and provide a holistic care, knowledge about older persons´ experience of living with long-term health problems as a whole is addressed in this study.

In order to define long-term and complex health problems, a variety of concepts, such as disease, illness and multimorbidity are used in the research literature. One of the most common term in the research literature in this context is multimorbidity, usually defined as the simultaneous occurrence of two or more diseases in one person (Marengoni et al., 2011). Multimorbidity increases significantly with age (Afshar et al., 2015; Gupta, 2016) and the most common state for 65–70% of 75-year-old persons is to have at least two chronic diseases (Abad-Díez et al., 2014; Fratiglioni et al., 2010). The predominant disabilities affecting older persons partly arise from age-related issues such as sensory impairment, related to vision and hearing (Fratiglioni et al., 2010; GBD 2015, DALYs and HALE Collaborators, 2016; WHO, 2015) and walking difficulties (GBD 2015, DALYs and HALE Collaborators, 2016; Meinow et al., 2015; WHO, 2015), and also partly from noncommunicable diseases. Common diagnoses in older persons are heart diseases, chronic respiratory disorders (The National Board of Health and Welfare, 2018; WHO, 2015), stroke, cancer, dementia (WHO, 2015), depressive disorders, osteoarthritis (GBD 2015, DALYs and HALE Collaborators, 2016) and diabetes (GBD 2015, DALYs and HALE Collaborators, 2016; The National Board of Health and Welfare, 2018). Impairment related to long-term pain, for instance, in back and neck through musculoskeletal conditions (Blyth et al., 2019; GBD 2015, DALYs and HALE Collaborators, 2016), dizziness, sleeping difficulties (Fratiglioni et al., 2010), fatigue and memory loss (Meinow et al., 2015) are examples of common health problems in older persons, not always viewed as a disease and labelled as a diagnosis (Fratiglioni et al., 2010; WHO, 2015). According to Toombs (1993), a disease is understood from a medical perspective as an objective and measurable phenomenon and differs from the patients’ subjective experience regardless of its manifestation in terms of a particular disease state, described as an illness. Health problem as a concept will be used in this study since it is a term that includes both the objective (disease, multimorbidity) and subjective (illness) perspectives. Long-term health problems in this study are defined as health problems (self-reported and defined by the older person) that have remained for at least 6 months (persistent or regularly recurring) and influencing the older persons’ daily living.

In Sweden, older persons with long-term health problems convey that they have both less confidence in and less access to healthcare that meets their needs compared to opinions from the population in general (The National Board of Health and Welfare, 2015). Together with high health care utilization and costs, functional impairment and poor quality of life are major consequences for older persons living with different health problems (Hallgren & Aslan, 2018; Hallgren et al., 2016; Marengoni et al., 2011; WHO, 2015). Research focusing on older persons living with long-term musculoskeletal pain at home revealed that they felt forced into learning to live life with pain on their own and coached themselves through daily life (Gillsjö, 2012; Gillsjö et al., 2013). Older persons living with post-stroke impairments and dementia revealed unnecessary suffering caused by caregivers due to lack of knowledge and understanding of the patient’s lifeworld; this caused feelings of insecurity, loneliness and alienation (Svanström et al., 2013). The lack of individual guidance and support, which is highlighted in previous research, is a problem that needs to be acknowledged in the healthcare system of today. In order to gain a deeper understanding of older persons living with long-term health problems and their needs, a transformation of health systems is required away from disease-based curative models and towards a holistic perspective to meet older persons’ complex needs of care when living with long-term health problems, based on the individual’s entire life situation in its own context. This includes all existing health problems and not just on the basis of parts, or occurrence of isolated health problems (Afshar et al., 2015; Berglund, 2011; Blyth et al., 2019; Ekman et al., 2011; Fortin et al., 2007; Gillsjö, 2012; Hallgren, 2016; Marengoni et al., 2011; Summer Meranius, 2010; Summer Meranius & Josefsson, 2017; WHO, 2015). Since ageing is often accompanied by various and often multiple health problems, it is significant to focus on how the long-term multiple health problems influence older persons’ daily living instead of focusing on the disease itself. This holistic approach is important, especially when designing and implementing interventions in health and social care.

### The STRENGTH intervention

Reflective STRENGTH-giving dialogue (STRENGTH) (Gillsjö & Berglund, 2014) is a method that is ontologically and epistemologically grounded in a lifeworld perspective (Heidegger, 2008; Husserl, 1989; Merleau-Ponty, 2002) and has been developed to meet the holistic need, based on research findings by Gillsjö (2012) and Berglund (2011). STRENGTH can be viewed as a way to apply person-centred care (Ekman et al., 2011) since a relationship evolves in which the person’s life situation as a whole comes to the forefront, and not the disease itself. STRENGTH has been used in a previous intervention study consisting of weekly dialogues during a period of 4 months with older persons living with long-term musculoskeletal pain (Berglund et al., 2016). The STRENGTH intervention will be used in the provision of healthcare in this present study, in order to meet the older persons’ needs in the often complex situation of living with multiple health problems. This initial interview study is needed to gain a deeper understanding about the participating older persons’ experiences of living with multiple long-term health problems and to collect baseline data in order to facilitate the evaluation of the effect of the STRENGTH intervention.

## Aim

The aim was to describe the phenomenon of living with long-term health problems that influence the daily living, from the older persons´ perspective.

## Method

The theoretical foundation in this study is the lifeworld perspective. According to Merleau-Ponty (2002), human beings perceive the world through the lived body. It is from the body that the person understands himself, others, and the world. The lived body is biological, thinking, feeling and acting at the same time. Living with long-term health problems entails a body that changes and this, in turn, affects the access to and the interaction with the surrounding world.

According to Husserl´s philosophy (Husserl, 1970a/1900), lived experiences are related to the natural attitude which is consequently basically unreflected. The lifeworld can be examined and conceptualized through reflection. An awareness of phenomena can be reached and available for analysis instead of just being taken for granted. Through reflection, new versions and a deeper understanding of lifeworld experiences are discovered and reconsidered (K. Dahlberg et al., 2008)

In this study, the phenomenon was described according to the Reflective Lifeworld Research (RLR) approach by K. Dahlberg et al. (2008), developed within traditions of phenomenology (Husserl, 1989; Merleau-Ponty, 2002). The overall aim of RLR is to gain an expanded and deepened understanding of a phenomenon by describing and clarifying lived experiences. In RLR, openness is used as a guiding tool for the methodological work. This entails the researchers understanding, and in particular, the pre-understanding, being bridled in relation to the phenomenon that is studied. Such openness involves the researchers not allowing theories or other preconceptions to affect their understanding. Bridling thus means striving towards a scientific reflective attitude, which entails being sensitive to the phenomenon and its meanings, with the particular aim of not making indefinite meanings definite too quickly (H. Dahlberg & Dahlberg, 2003).

### Participants and data collection

The study was conducted in community-based health and social care in one community in the western region of Sweden. The participants were recruited according to a strategic sampling procedure in regard to geography in order to avoid the professional relationship between the participants and the healthcare professionals providing the STRENGTH intervention. A development manager asked healthcare professionals (nurses, occupational therapists, physiotherapists) to identify older persons that met the inclusion criteria. They reported name and contact details back to the development manager. In total, a number of 52 persons were identified that met the inclusion criteria: 65 years or older, living with long-term, persistent or regularly recurring, health problems for at least 6 months and receiving support from the home care services. Additionally, they had to be able to understand and answer questions in Swedish. The older persons that were identified by the healthcare professionals were contacted by the development manager or the healthcare professionals themselves by telephone or personal contact to provide oral and written information about the study. Older persons who consented to participate or wanted more information and agreed to have their contact details given to the researchers, were contacted by the first author. Of these 37 older persons, 34 wanted to be included in the study. Among the participants (n = 34), 23 were women and 11 men with a mean age of 86 years (range, 74–96 years). The participants lived alone (n = 32) or with their spouses (n = 2) in ordinary housing (n = 24, 10 in houses and 14 in apartments), independent living in senior housing (n = 5) or assisted living facilities (n = 5). The participants’ self-reported long-term health problems, which varied in number from three to 16 and examples of health problems were dizziness, mobility problems, tiredness, fatigue and pain.

The lifeworld interviews were carried out as dialogues according to the principles of RLR (K. Dahlberg et al., 2008). The interviews were conducted by the first author in the participants’ homes at their request, recorded and lasted an average of 34 min (range 9–84 min). The opening question—“Would you please tell me what it’s like for you to live with long-term health problems?” was asked to direct the participants’ intentionality towards the particular phenomenon. The interviewer was required to be restrictive and not take anything for granted. Further intentions were to stay curious, open-minded and pliable to the phenomenon by questioning and responding to the participant in order to support and encourage the participants’ attempts to reflect on the phenomenon. Follow-up questions (e.g., “Could you tell me more?”, “How did you experience it?”) were asked to clarify and to gain a deeper understanding of the phenomenon.

### Phenomenological lifeworld analysis

The analysis (Dahlberg, 2006) aimed to describe the core aspects of the phenomenon on an abstract level, i.e. the essence, and all its variations and nuances on a more concrete level, called the constituents. The data, consisting of lifeworld descriptions, were analysed by the researchers according to a tripartite structure with a movement between the whole—the parts—the whole. An open, reflective attitude in a bridling manner was conducted through the process, according to the principles of RLR (K. Dahlberg et al., 2008), which are further illuminated below.

The recorded interviews, including non-verbal information such as silence, were transcribed verbatim by the first author. The analysing process continued using open-minded reading and re-reading the interviews to become familiar with the whole content. An effort was made to keep the pre-understanding in the background by searching for “otherness”, and not only confirming what was already known. After this initial reading, the researchers continued the descriptive analysis (K. Dahlberg et al., 2008) by identifying units of meaning: smaller segments (word, a sentence or a longer piece of text), connected to the phenomenon. Questions were asked to the text, for example, “How and in what way is living with long-term health problems influencing your daily life shown in the described situation?” The subsequent reading was focused in order to understand the meaning of every unit (Van Wijngaarden et al., 2017), carefully not taking meaning for granted, in an attitude of carefulness and reflection. One question that was asked in the process of understanding was if the meaning of the text could be understood in any other way. This question was asked repeatedly to deliberately slowdown the understanding and increase the reflection in ways that support openness and sensitiveness in understanding the phenomenon. The movement between the parts and the whole in the analysis can according to RLR (K. Dahlberg et al., 2008) be described as working in the vacillation between the two terms figure and background to reveal essential meanings and patterns. This work entails the movement between considering each meaning in the forefront as a figure against the background of the whole. For example, how could the meaning of uncertainty be understood in relation to the entirety? Could the meaning entail something else? Meanings that seem to belong to each other were then put together and labelled in clusters, which is to be seen as a temporary pattern. Rereading all clustered meanings were a help for the researchers to view the pattern that formed the essential meanings of the phenomenon living with long-term health problems influencing daily living as experienced by older persons on an abstract level. When the essence of the phenomenon was described, the analysis continued in order to describe the constituents, i.e. all the variances and nuances of the phenomenon on a more concrete level. The essence and the four constituents, illuminated with quotes, i.e. the new whole, are presented in the results.

## Ethical considerations

The study was approved by the Regional Ethical Review Board of Gothenburg (No. 295–17) and carried out in accordance with the principles outlined in the Declaration of Helsinki (World Medical Association, 2013). Oral and written informed consent was obtained from each participant after they were provided with both written and oral information about the aim of the study, i.e. that the participation was voluntary and that confidentiality would be maintained.

## Results

The essence of living with long-term health problems that influence the daily living, from the older persons´ perspective, is to be seen as living in a diminishing world. It consists of living with insecurity and unpredictability in terms of access to the world. This access is influenced by the body’s ability, which varies over time and in relation to the intensity of each individual’s various health problems. Living with long-term health problems comprises becoming and being in need of support in order to cope with daily life and to gradually be deprived of decision-making in one’s own life. In this diminishing world, there is a strive towards finding new meaning in life with long-term health problems. A further description of the phenomenon is followed by its constituents: To live in uncertainty and not be able to trust one’s own ability; To be deprived of decision-making; To be dependent on support in daily life; and Striving to maintain meaningfulness in daily living.

### To live in uncertainty and not be able to trust one’s own ability

Living with long-term health problems is experienced by older persons as living in uncertainty about one’s own abilities. Living with several health problems means living with a body in constant change; these changes make the body erratic, which affects access to the world.

The health problems are difficult to influence. One by one they are forced, unwanted and unable to reverse. “Yes, one always has to live with the problems and they keep coming the older I get”. The complexity of living with several simultaneous health problems is that its intensity and impact on daily life can vary from time to time, from day to day but also during the day. “One day I can have more troubles with my legs and one day it is my hands. Other days are different. Sometimes it is better and sometimes it is worse”. The variation of health problems can mean that the older person feels unsure and does not trust their own ability, which creates uncertainty and unpredictability. The health problems affect each other and can, in turn, create new health problems. An older woman with a risk of falling describes it as follows: “Yes, it’s all connected one can say as I have bad muscles in my legs, the feet are weak which gives me bad balance … after all, I have broken many bones and it’s a vicious circle”.

Difficulties in predicting the overall impact of health problems on daily life limit the possibilities of planning activities in advance. For fear of what may happen, the older person does not expose him/herself to activities that he/she does not believe they can cope with. An older woman describes this fear as no longer daring to be brave enough or making movements on her own as she is afraid of falling, which in itself entails a risk of sedentary sitting with further deterioration as a result.

Living with long-term health problems is experienced as living with a threatened existence where life is limited and the end of life might be in the near future. Life is fragile and thoughts of the transience of life increase. An older man describes how he lives with this thought in order to be prepared for the health situation and to sudden life changes. “And as I said, there is an expression in Latin, alt trumpus paratus, be prepared for both of them”. An ageing body is not expected to cope with everything, an older woman describes that as her organs are failing, due to old age and illness, there is no help for her and all she can do is “wait for the end”. Living with this insight creates different thoughts about the future. Concern is expressed about what the health problems and a possible deterioration will bring, while others express a calmness about the future. With age, life is expected to be increasingly limited by health problems, one accepts the situation, demands less of life and does not plan so much for the future. An older man who has suffered two heart attacks; “If I get another one as powerful as the one I had the last time, I think it would kill me. But I’m not thinking about that. I take each day just as if I should live a very long time. So it may come when it comes”. A gratitude for having reached an advanced age is expressed and with the certainty that further health problems will possibly arise, life is lived one day at a time.

### To be deprived of decision-making

Living with long-term health problems is experienced by older persons as if the possibility of making their own choices is impaired, because others such as relatives and healthcare providers have views on what is best for them and how they should live their lives. Due to health problems, the body is unpredictable and constantly changing. This tends to affect the trust of others in the ability of the persons to live autonomously and prevents participation in decisions that concern themselves.

The older persons sense that close relatives are afraid that something might happen and thus take over the decision-making as they need to feel secure. The noted fear seems to increase in parallel with the increasing health problems. An older woman expresses the following regarding her children’s decision on care efforts: “It’s not me but the children who wanted me to have the help as they would feel a little safer then when they are far away”. The opinions regarding decisions about what is the best are divided, and costly efforts are made, for example, the handling of medication and support in the shower, which the older persons, in fact, consider to be able to do themselves. It is also due to decisions regarding the ability to drive a vehicle or not, that the older person’s right to make their own decisions is removed. “I had to stop driving my car and I am not even allowed to ride a bike, but that was the family’s decision”.

The healthcare providers prevented participation in the decision-making. For example, the older persons express how healthcare providers decided how furnishings should be placed in the home, so that the working environment is good for them, or that more help is advocated than what the older person him/herself considers necessary. It is described that healthcare providers decide that a wheelchair is to be used instead of a walker even though the older person can still walk. “They have started to put me in a wheelchair now. I try to walk but they say no we take the wheelchair”.

The possibility of resettlement is also an issue where decision-making is being made by others. An older man describes how health-care providers initiated a care plan where they tried to persuade him to move from his house to an assisted living facility. “They were here a while ago and everybody wanted me to move. Three staff were here trying to convince me but I want to stay at home“. Despite health problems, the older persons considered it valuable to manage as much as possible in everyday life as far as it goes. “As long as I know I can handle it, I want to do it“. The feeling of independence holds one ”eager” and ”active” and is important for gaining power and motivation in order to live on. On the other hand, situations are described in which healthcare providers, on the contrary, have too high a confidence in the older person’s ability and thus very high demands. An older woman describes how she is expected to be able to both wash herself and cook her food, but there is not enough strength to perform all the daily tasks. Not being involved in decisions creates a feeling of not having power over their own lives.

### To be dependent on support in daily life

Living with long-term health problems entails becoming dependent on, for example, close relatives, assistive devices, medicines and healthcare providers in order to cope with daily life. It is valuable that the support does not intrude upon the feeling of independence. The dependence affects the possibility of spontaneity to do what is valuable in life, where both health problems and conditions for planning and structure in everyday life vary.

Support, on the one hand, facilitates the independence of the everyday life as it can compensate for some ability to a certain limit. Being dependent, on the other hand, is experienced as a loss of independence and a fear when one is dependent on this support. The health problems can pose difficulties when it comes to learning or using assistive devices and household appliances, which creates dependence on others in daily life. An older woman describes how she has become dependent on interventions from healthcare providers several times daily as she received a new kind of insulin cannula which she cannot handle due to reduced hand motor skills. “The new ones are too difficult for me to use as I have no power in my right hand”.

There is a conflict between not wanting to be a burden to others and having to ask for support. There is concern about being a burden and a feeling of not wanting to cause trouble, hence it is found that opportunities are waived with regard to asking relatives for support. “It is so hard for me to take time from other people’s lives. So, I find it very difficult to demand and ask for help”. Having to be someone else’s load to carry often leads to a guilty conscience but gratitude for support is described as the health problems are increasingly affecting daily life.

A trusting relationship with supportive healthcare providers creates security and inspires confidence in the older persons allowing them to want to talk about their life and health problems. To be understood as a unique individual is valuable. Being able to talk to healthcare providers about one’s life when living with health problems lightens the load, facilitates motivation and gives power and willingness to come back after temporary weaknesses. An older woman who returned to her home after her third surgery was given new strength when she met the welcoming of healthcare staff at the gate. “Yes, it felt so life giving. It felt like a warm embrace. One takes charge of everything and thinks: Sorry, but god damn it, let’s get this thing rolling. And so it was”.

The older persons explain that they experience how health-care providers pay attention to the specific health problem and give advice and solutions how life with health problems can be facilitated, without paying attention to the older person’s whole and unique situation. Situations are described where the conversation is not prioritized and about the lack of support on how life is handled with all the health problems, which leads to the individual feeling abandoned and lonely in life. “The district nurse, she could talk to me but she doesn’t. Just talking about what is wrong with me, but she doesn’t care. She should sit down and chat a little”. Lack of knowledge is experienced about the cause, treatment and development of the health problems, the effect of drugs and how the health problems are related. The older persons’ experiences that that advanced age influences the healthcare providers in such a way that the care is concentrated on one health problem at a time and only solve those issues in the short term. “When I say I feel bad, they say there is nothing to do for me because I am old”. While support is described to compensate for losses in daily life, there is a lack of support for the complex whole, such as living with long-term health problems.

### Striving to maintain meaningfulness in daily living

Living with long-term health problems means living in a changing world. The quality and content of life are limited and health problems contribute to a forced solitude. Although the health problems affect daily life, there is a pursuit for meaningfulness in life. It is valuable for the older persons to have activities which they themselves find meaningful. The joy of living is described as important for motivation, to feel hope and not to give up.

On one hand, a sense of loss of how life once was is described. The health problems limit the possibilities for what previously made sense in life such as walking, going to the movies, restaurants and concerts, accessing driving vehicles, visiting the grandchildren and the cemetery. “I would like to bake bread. I always did that and cookies … what fun it was. I can’t stand up for that long because of my back. So, no point in thinking about it”. Even external factors such as the seasons affect the possibility of activities. Meaningful activities are excluded when the experience is no longer the same. An older woman describes how, due to impaired vision, she prefers to stay at home rather than to accompany her son to the grocery store. The impaired experience and ability mean that the older person, against his will, becomes more and more home bound. ”I feel trapped sometimes … in my soul you could say. I sit here and look out and see everyone who is out and about and I can’t. And you have to live with that, but it’s not easy”.

Meaningful activities with others are also limited by not wanting to show the shortcomings that health problems bring. An older woman expresses that she no longer wants to eat with others as her impaired vision entails difficulties with her table manners. Meeting other older persons and seeing how they handle life with health problems increases motivation to live on. The health problems make it more difficult to keep up to date about what is happening in society, which leads to a feeling of isolation and exclusion. “I can’t watch TV, I can’t read books or a newspaper. Yes, life is very slow”. The contents of life are limited and the world diminishes as the older persons find it difficult to maintain the contacts and activities that are meaningful to them.

The older persons miss the lives they once had, but also describe it as an endeavour to find new meaning in life despite the health problems. An older woman describes her own responsibility for finding meaning in life; “What I can no longer enjoy, I myself must create and find new entertainment”. Social media over the Internet, such as Facebook, as well as Google Earth and games on computers etcetera, give access to the world outside of one’s own home and are described as a pastime and meaningful occupations. The possibility of using these media also gives the opportunity to watch TV programmes at times that fit into their own everyday life. They find their own strategies to facilitate and manage everyday life with health problems by testing, thinking in new ways and learning through mistakes. By listening to the body’s needs and signals, an understanding of the body’s ability and limitation is obtained. Having a target image is perceived to be a support in the endeavour to maintain meaning in life. An older woman expresses how this target image has been a support to get back after a hip operation that has caused severe pain. “I must try. Not just think there’s no point, there’s nothing more to do. If I think like that, I’ll never get back to a life and I can’t and don’t want to think like that. One must try and the goal is to come back”.

Characteristics such as persistence, gratitude, courage and curiosity are described as facilitating the pursuit of meaning in life with long-term health problems.

## Discussion

The results show that living with long-term health problems that influence the daily living, from the older persons´ perspective, is experienced as living in a diminishing world with an unpredictable body that constantly changes. This can be viewed in light of Merleau-Ponty (2002) claiming that living with a body in change affects the access to and the interaction with the world. The results of this study show that this affected access to the world contributes to the risk of a sedentary lifestyle and reduced social network resulting in further deterioration. In addition, the present results show that living with long-term health problems is experienced as a situation of being gradually deprived of decision-making in one’s own life. Although the WHO (2015) declares that older persons must be included as active participants in their own care and in managing their own health, the results in the present study reveal that relatives and healthcare providers are afraid that something undesirable might happen to the old person they are caring for. They take over and make decisions about the older person’s needs to increase the feeling of being safe, but the results show that this gives the older person a feeling of being deprived of autonomy. In line with the results of this study, previous studies (Årestedt et al., 2018; Mazer et al., 2014) have found that family members base the conversations with healthcare providers on their own beliefs and consider it as their mission and duty to speak for and to see to the older person’s interests. The families explained their actions and stated how illness in older persons was not taken seriously by healthcare providers. From an older person’s perspective, Sundström et al. (2018) describe how, in health and social care planning meetings, an asymmetric relationship in decision-making made the older persons feel they could not always speak for themselves, they were not involved and sometimes felt unable to defend their own interests. Årestedt et al. (2018) found that persons will illnesses sometimes preferred to meet healthcare providers by themselves to prevent family members from “taking over” conversations. Even though it is because of genuine concern that others make decisions in the older person’s place, it is shown in the present study that it is important for the older persons to feel independent in order to gain power and motivation to carry on. Increased knowledge, awareness and understanding of how older persons perceive their situation is required from both relatives and healthcare providers in order to encourage older persons and maintain their sense of autonomy in life, when living with long-term health problems.

As a healthcare provider assessing healthcare needs, it is important to focus on the impact of health problems on the older person’s life, instead of only considering the specific illnesses experienced (WHO, 2015). The results, however, show that in conversations with an older person, the healthcare providers focus on solutions and give advice regarding how life with health problems can be solved rather than listening to the older person’s story. This is similar to findings in the research in which older persons living with chronic heart failure express how healthcare professionals focus on explanations related to the illness rather than on concerns about issues affecting everyday life (Östman et al., 2015). Furthermore, the results in the present study reveal a lack of support concerning how life is handled with all the health problems; this leads to a feeling of abandonment for the older person. Consequently, this indicates that the healthcare for older persons needs to be improved in order not to cause harm but instead improve the feeling of well-being. According to the concept of sustainable development, promoting well-being for individuals at all ages is essential (United Nations, 2019; WHO, 2013). There is a need to tailor individual holistic care in the provision of healthcare to promote and preserve health and well-being (Gillsjö et al., 2013). Within a perspective of lifeworld-led care (K. Dahlberg et al., 2009), where a holistic contextuality of lifeworld experience comes to the forefront, patients’ well-being can be supported when healthcare professionals listen to the patients’ understanding and knowledge of their experience of living with different health problems and the impact on their lives. Listening to the patient can be accomplished by healthcare providers simply by having the courage to remain present in the situation and by paying close attention to the needs of the patient (Andersson et al., 2015). The results in the present study show that there is a need for healthcare providers to focus on developing a relationship that is characterized by an approach that mediates trust and support, so that the older persons feel confident to talk about life with health problems. A report from The National Board of Health and Welfare (2018) reveals that many older persons feel safer when receiving support and help from healthcare providers they recognize and have come to know. Despite the fact that the number of employees in Swedish municipal healthcare has increased, staff continuity in the care of older persons has deteriorated over the past 10 years. It is also noteworthy that healthcare is the primary subject in all occupational areas where there is a shortage of competent staff. The World report on ageing and health (WHO, 2015) states the importance of healthcare providers having knowledge in issues common to old age in order to provide good care, which is not always the case. This development is contrary to the factors needed that would contribute in developing a trusting relationship between the older person and the healthcare provider through dialogue. Dialogue is needed in order for healthcare to be person-centred, which means that care is planned, organized and implemented based on an understanding of the patient’s lifeworld and relationship to illness, treatment and desired care (K. Dahlberg & Ekman, 2017; Ekman et al., 2011). In order to provide person-centred care, the conditions for having dialogues in which the older person’s lifeworld comes to the forefront must be improved. Continuity and competence could, therefore, be important aspects to consider in order to meet the needs of older persons living with long-term health problems in the health-care system.

Göransson et al. (2017) found that relationships and social activities are valuable for older persons’ perceived health. To increase interaction with the surrounding society, information technology (IT) was found to be used by the older persons in the study; this appears to provide a new meaning in life for those with long-term health problems. Using IT proved to decrease loneliness and social isolation in older persons (SBU, 2017b). However, a need for education may exist which need to be considered in the provision of care. The health problems can also entail difficulties in using IT and this needs to be taken into consideration. The results in the present study show that older persons may need support in order to find a new meaning in life when living with long-term health problems. According to the World Health Organization (WHO) there is extensive literature on the physical and mental processes of old age but determining what older persons really need and what is important for them needs to be explored (WHO, 2017). From a caring science perspective, it is possible to experience health and well-being despite health problems (K. Dahlberg et al., 2007, 2009). Through reflection, awareness of one’s actual situation is attained and responsibility and actions could be taken for what can or cannot be changed (Berglund, 2014). Health-care professionals have an important role to play in supporting this reflection. Oriented towards meaning in life, STRENGTH (Berglund et al., 2016) was shown in a previous study to support reflection through dialogues. In older persons living with long-term musculoskeletal pain, STRENGTH was experienced as a continuous and trusting relationship that increased a sense of health and well-being as it alleviated pain and broke the loneliness. Since older persons have a great need for healthcare the need for care will increase (The National Board of Health and Welfare, 2018; WHO, 2006, 2017). An important part of this is to ensure that care is adapted to various groups’ different needs in the growing populations (WHO, 2015). Maintaining and finding new meaning in life seem to be valuable in overcoming the negative aspects of living with long-term health problems and the feeling of living in a diminishing world. The present results address healthcare providers’ important role in ensuring and supporting older persons to enhance meaning in life, despite health problems.

### Methodological considerations

Van Wijngaarden et al. (2017) present a philosophical foundation for phenomenological evidence by exploring the notions of objectivity, validity and generalizability in terms of openness, meaning and essence. Reaching objectivity is about bridling one’s understanding as a researcher, as well as constantly remain open to the phenomenon in order to obtain new results and not only confirm what is already known to the researcher (K. Dahlberg et al., 2008). Validity is a concept that commonly describes how well the study measures what it intends to measure. In RLR, validity is associated with the research being meaning-oriented. Including available background information about the informants means that readers can compare and draw their own conclusions about generalizability to other contexts (Van Wijngaarden et al., 2017). Background information that is described in the study regards the informants’ age, gender, family relationship and housing situation. One consideration may be how much information needs to be published for the reader to be able to draw conclusions about generalizability. In the 34 interviews with older persons, meaning units were distinguished in order to identify the meaning of the phenomenon studied. The data were approached with an openness, curiosity and a simultaneous commuting between particularity and generality, trying to ensure that the meaning units responded to the phenomenon. When validity is associated with meaning (Van Wijngaarden et al., 2017), the researcher needs to know the difference between content and meaning. It is the meaning of the phenomenon that should be in focus rather than describing what the informant expresses by its content. This process was questioned and problematized as all authors have participated in the analysis. Two of the authors have also previous experience of this particular type of analysis. The strength of the approach, compared to other qualitative methods, and what increases the generalizability, is that the results are presented on an abstract level in the form of an essence with its constituents, which characterize the phenomenon (Van Wijngaarden et al., 2017). The clear phenomenon orientation contributes to the result being generalized, i.e. that the result may apply to more than those included in the study (K. Dahlberg et al., 2008). An essence is not considered to be fully explored or described since meaning changes depending on how the lifeworld changes (Dahlberg, 2006); this means that the need for the number of informants is difficult to comment on. Instead, it is crucial that the data is rich in the meaning of lived experiences of the phenomenon. Likewise, the sample should achieve a variation of experience in order for the result to be generalized (K. Dahlberg et al., 2008). Furthermore, the data is influenced by how the lifeworld interviews were conducted. The participants could influence time and place of the interviews, however disturbing factors such as telephone calls, visits from the social- and health-care services and other activities interrupted the collection of data. This can be seen as limitations in the study since it might have influenced the interaction and ability to remember and reflect, resulting in a subsequent effect on the data collected. During the data collection, the compliance to the phenomenon was developed by being curious, asking supplementary questions, not taking anything for granted by searching for “otherness”, and not only confirming what was already known. The co-writers listened to the recorded interviews to ensure that they contained the depth and meaning required for the approach and to confirm that the phenomenon was in focus. The older persons’ vulnerability had to be kept in mind during the interviews. The informants were a frail group; in some of the interviews, this was noticed when the participants became tired and asked to finish the interview in advance, for example, one of the interviews only lasted for 9 min. This may also have to do with the order in which data were collected in the intervention study. Quantitative data were collected before the interviews, which should be seen as a limitation in the study. However, even short interviews can contain rich descriptions of the phenomenon. It is also of importance that vulnerable and frail persons´ voices are heard and included in interview studies. A concern that the informants wanted to declaim their participation in the project if the interviews were too demanding may also have affected the length and depth of the data material and should be seen as limitations. Moreover, differences related to the number of health problems as well as the housing situation have not been taken into consideration in the analysis.

## Conclusions

The results show that life with long-term health problems entails living in a diminishing world. Being affected by several health problems entails not being able to trust one’s own ability and the freedom to make decisions of your own is deprived by relatives and healthcare providers. Opportunities to talk about how to deal with life when living with health problems, as well as existential issues, are important for the older person’s well-being. Support that preserves self-determination and stimulates the implementation of small and large life projects that bring joy and meaning in life is valuable for the older person; however, this is rarely met in the provision of health and social care. The prerequisites for dialogues in the context, in which the older person’s lifeworld comes to the forefront must be improved. Consequently, the results address the need of the extended individual and holistic guidance and support in living with long-term health problems, in order to increase the older person’s sense of well-being and meaning in life.
